# Notice

**Published:** 1871-05

**Authors:** T. D. Fitch

**Affiliations:** Permanent Sec’y Ill. State Med. Soc.


### *Illinois State Medical Society.*

Office of the Permanent Secretary, 296 W. Monroe street, Chicago, Ill., April 19, 1871.

The twenty-first annual session of this Society will be held in the city of Peoria on the third Tuesday (16th) in May, 1871, at 10 A. M.

The following Committees are expected to report:

On Practical Medicine—Dr. H. A. Johnson, of Chicago, Chairman.
On Surgery—Dr. E. Andrews, Chicago, Chairman.
On Obstetrics—Dr. De Laskie Miller, Chicago, Chairman.
On Drugs and Medicines—Dr. E. Ingalls, Chicago, Chairman.
On Criminal Abortions—Dr. W. H. Byford, Chicago, Chairman.
On Ophthalmology—Dr. H. H. Roman, Springfield, Chairman.
On Phthisis Pulmonalis—Dr. S. W. Noble, Bloomington, Chairman.
On Otology—Dr. S. J. Jones, Chicago, Chairman.
On Criminal Insanity—Dr. A. McFarland, Jacksonville, Chairman.
On Idiocy—Dr. C. T. Wilbur, Jacksonville, Chairman.
On Medical Uses of Carbolic Acid—Dr. N S Davis, Chicago, Chairman.
On Cholera Infantum—Dr. D. W. Young, Aurora.
On Membranous Croup—Dr. D. Prince, Jacksonville.
On Gleet—Dr. R L. Higgins, Vandalia.
On Results in Fractures—Dr. G. W Phillips, Dixon.
On Intestinal Affections of Infants—Dr. T. D. Fitch, Chicago.
On Diabetes—Dr. W. D. Sterling, Ionia.
On Perineal Section—Dr. E. Powell, Chicago.
On Duties of the Profession to State Med. Soc.—Dr J. S. Whitmire, Matamora.
On Causes of Mortality after Amputation—Dr. R. N. Isham, Chicago.

Medical Societies are entitled to one delegate for every five of its regular resident members, and one for every additional fraction of more than half this number.

The Faculty of every regular Medical College is entitled to two delegates. Every chartered or municipal hospital is entitled to one delegate.

Any respectable physician, unable to attend as a delegate or permanent member, may attend as a *member by invitation*, on the recommendation of the Committee of Arrangements.

*Railroad Arrangements.*—Arrangements have been made with all the railroads leading to Peoria—to return delegates and permanent members for one-fifth fare on presentation at the ticket office of a certificate signed by the Secretary of the Society, except the Chicago, Rock Island and Pacific, with which company we have the following arrangements: Any delegate or member expecting to travel over this road to and from the Society, must have an order, which he can obtain from me, before leaving home, which on being presented to the ticket agent at any station on the road, will entitle the holder to an excursion or round trip ticket, at sixty per cent. of the regular fare.

I shall send to every member of the Society, that will probably go over this road, an order, which if they do not wish to use, they may hand to any delegate or member that desires to go—or return to me.

T. D. Fitch, M.D.,

*Permanent Sec'y Ill. State Med. Soc.*

